# Acute monoarthritis in young children: comparing the characteristics of patients with juvenile idiopathic arthritis versus septic and undifferentiated arthritis

**DOI:** 10.1038/s41598-021-82553-1

**Published:** 2021-02-09

**Authors:** Marion Thomas, Stephane Bonacorsi, Anne-Laure Simon, Cindy Mallet, Mathie Lorrot, Albert Faye, Glory Dingulu, Marion Caseris, Ivo Gomperts Boneca, Camille Aupiais, Ulrich Meinzer

**Affiliations:** 1 Department of General Pediatrics, Pediatric Internal Medicine, Rheumatology and Infectious Diseases, National Reference Centre for Rare Pediatric Inflammatory Rheumatisms and Systemic Autoimmune Diseases RAISE, Robert Debré University Hospital, Assistance Publique-Hôpitaux de Paris, 75019 Paris, France; 2 Institut Pasteur, Biology and Genetics of Bacterial Cell Wall Unit, Paris, France; 3 CNRS UMR2001, Paris, France; 4 INSERM, Equipe Avenir, Paris, France; 5 Université de Paris, Paris, France; 6 Department of Microbiology, Robert Debré University Hospital, Assistance Publique-Hôpitaux de Paris, Paris, France; 7 Pediatric Orthopedic Department, Robert Debré University Hospital, Assistance Publique-Hôpitaux de Paris, Paris, France; 8 Pediatric Department, Division of Infectious Diseases, Armand Trousseau Hospital, Assistance Publique-Hôpitaux de Paris, Paris, France; 9 Pediatric Emergency Department, Jean Verdier Hospital, Assistance Publique-Hôpitaux de Paris, Paris 13 University, Bondy, France; 10 INSERM, U1138, Equipe 22, Centre de Recherche des Cordeliers, Paris, France; 11 Centre de Recherche sur l’inflammation, UMR1149 INSERM et Université de Paris, Paris, France

**Keywords:** Biomarkers, Medical research, Rheumatology

## Abstract

Acute arthritis is a common cause of consultation in pediatric emergency wards. Arthritis can be caused by juvenile idiopathic arthritis (JIA), septic (SA) or remain undetermined (UA). In young children, SA is mainly caused by *Kingella kingae* (KK), a hard to grow bacteria leading generally to a mild clinical and biological form of SA. An early accurate diagnosis between KK-SA and early-onset JIA is essential to provide appropriate treatment and follow-up. The aim of this work was to compare clinical and biological characteristics, length of hospital stays, duration of intravenous (IV) antibiotics exposure and use of invasive surgical management of patients under 6 years of age hospitalized for acute monoarthritis with a final diagnosis of JIA, SA or UA. We retrospectively analyzed data from < 6-year-old children, hospitalized at a French tertiary center for acute mono-arthritis, who underwent a joint aspiration. Non-parametric tests were performed to compare children with JIA, SA or UA. Bonferroni correction for multiple comparisons was applied with threshold for significance at 0.025. Among the 196 included patients, 110 (56.1%) had SA, 20 (10.2%) had JIA and 66 (33.7%) had UA. Patients with JIA were older when compared to SA (2.7 years [1.8–3.6] versus 1.4 [1.1–2.1], p < 0.001). Presence of fever was not different between JIA and SA or UA. White blood cells in serum were lower in JIA (11.2 × 10^9^/L [10–13.6]) when compared to SA (13.2 × 10^9^/L [11–16.6]), p = 0.01. In synovial fluid leucocytes were higher in SA 105.5 × 10^3^ cells/mm^3^ [46–211] compared to JIA and UA (42 × 10^3^ cells/mm^3^ [6.4–59.2] and 7.29 × 10^3^ cells/mm^3^ [2.1–72] respectively), p < 0.001. Intravenous antibiotics were administered to 95% of children with JIA, 100% of patients with SA, and 95.4% of UA. Arthrotomy-lavage was performed in 66.7% of patients with JIA, 79.6% of patients with SA, and 71.1% of patients with UA. In children less than 6 years of age with acute mono-arthritis, the clinical and biological parameters currently used do not reliably differentiate between JIA, AS and UA. JIA subgroups that present a diagnostic problem at the onset of monoarthritis before the age of 6 years, are oligoarticular JIA and systemic JIA with hip arthritis. The development of new biomarkers will be required to distinguish JIA and AS caused by *Kingella* *kingae* in these patients.

## Introduction

Juvenile Idiopathic Arthritis (JIA) is a heterogeneous group of diseases characterized by chronic inflammation of the joints of unknown origin during at least 6 weeks in children younger than 16 years of age^1–3^. It is a clinical diagnosis based on history and physical examination. JIA has different subtypes that are defined according to the number of joints involved in the first 6 months of the disease and extra-articular involvement. In European populations, the annual incidence rate for all forms of JIA ranges from 1.6 to 23/100,000^4^. In the first few days after onset of arthritis, particularly when only one joint is involved in young children, patients are often referred to the emergency departments for investigation of acute arthritis.

Acute arthritis is a common cause of visits to pediatric emergency departments. Several diseases can lead to the development of acute arthritis. Although JIA is the most common chronic rheumatic disease in children, it accounts for only a small fraction of patients presenting with acute arthritis at the emergency departments as many patients with JIA are oriented towards pediatric rheumatologists.

In children, septic arthritis (SA), also known as joint infection or infectious arthritis, is a frequent etiology of acute arthritis in children and usually occurs as a complication of bacteremia^5,6^. The overall incidence of acute SA is estimated to be 4–10 per 100,000 children in well-resourced countries, with a highest hospitalization rate before the age of 4 years, and knee and hip arthritis being the most common localization^7,8^. *K. kingae* (KK), a Gram negative bacteria, has been shown to be associated with bone and joint infection in young children, and is now recognized as the leading pathogen of this type of infection in children younger than 4 years of age, accounting for up to 80% of microbiologically confirmed cases^5,9,10^. The gold standard for the diagnosis of SA is isolation of the causative agent from joint fluid or blood, but this is not always possible^11,12^. A national study reported blood-borne bone and joint infections in a pediatric population, with the pathogen identified in only 28% of cases^13^. Molecular methods such as PCR of the 16S rRNA gene, followed by KK-specific real-time PCR, make it possible to detect KK in children with osteoarticular infection with very good sensitivity, but in "real life", results are often only available a few days after admission to hospital^9,10^.

In some cases, non-septic arthritis has other origins such as neoplasia, or other rheumatic and systemic inflammatory diseases, with acute arthritis being the opening presentation. Finally, arthritis remains of unknown origin in the absence of SA or JIA criteria and without any other specific diagnosis. Throughout this manuscript, these forms are referred to as undetermined arthritis (UA).

Involvement of two or more joints is rarely seen in SA^5^. However, in the case of monoarthritis, the differential diagnosis between SA and JIA occurring in young children before the age of 6 years (also called “early onset JIA”) can be challenging at an early stage. This is particularly true for arthritis caused by KK, the predominant pathogen of arthritis in young children, which is known to induce only mild clinical arthritis and biological inflammatory response^5,6^. The differential diagnosis between monoarticular early onset JIA and SA is essential because children with SA need urgent treatment, such as surgical joint irrigation (lavage/drainage) followed by intravenous antibiotics, to avoid infectious complications^14–18^. In contrast, children with JIA should be referred to a specialist for specific treatment, including non-steroidal anti-inflammatory drugs and/or intra-articular glucocorticoid injections and/or DMARDs and biological therapeutics^19–22^. Drainage and immobilization of the joint may delay appropriate treatment of JIA if misdiagnosed, resulting in further disease progression and increased joint erosion and disability^23–26^.

The aim of this work was to compare clinical and biological characteristics, length of hospital stays, duration of intravenous (IV) antibiotics exposure and use of invasive surgical management, in young patients (< 6 years) with acute monoarthritis with a final diagnosis of JIA, SA or UA.

## Methods

We included all successive patients aged from 3 months to 6 years, who underwent joint aspiration for acute arthritis at the University Hospital Robert-Debré (Paris, France) between 2015–2018. Patients were identified by screening the hospital database and medical records. Exclusion criteria were symptoms lasting ≥ 6 weeks, oligo- and poly-arthritis (≥ 2 joints), arthritis associated with osteomyelitis, a pre-existent diagnosis of JIA, a previous hospitalization for arthritis, another specific diagnosis not septic neither inflammatory, previous anti-inflammatory therapy.

A second data collection using the same inclusion and exclusion criteria had been carried out in the framework of a previous study between 2008 and 2009^27^. As the two cohorts had comparable patient profiles (supplementary data Table 1), we combined cohorts in order to increase the number of observations.

For all patients demographic and clinical data including duration of symptoms, localization of arthritis and presence of fever were collected. Biological data at the beginning of hospitalization were also recorded: hemoglobin, platelets, white blood cells and neutrophils counts, C-reactive protein (CRP), fibrinogen, pro-calcitonin (PCT), sedimentation rate, result of blood bacterial cultures. In synovial fluid, white blood cell counts and microbiological examinations were reported (direct microscopic examinations, bacterial culture, 16S RNA-PCR and KK-PCR if performed). Management of acute arthritis was also assessed: length of the hospital stays, treatment with antibiotics and its duration, surgical interventions (e.g. arthrotomy-lavage).

JIA was defined according to ILAR criteria^3^. This diagnosis was based on the follow-up visits with a pediatrician and/or a pediatric rheumatologist, after the initial hospitalization. SA was defined as arthritis with positive bacterial culture in synovial fluid and/or blood cultures or positive KK-PCR or 16S ribosomal-PCR in synovial fluid. UA was defined as arthritis not fulfilling the criteria of SA or JIA, without any other identified specific etiology.

The study was carried out in accordance with relevant national guidelines and regulations. The study was approved by the Institutional Review Board of Paris-North Hospitals, AP-HP (IRB no. 2013-84), the Comite de Protection des Personnes Nord Ouest III (2019-A02427-50) and the French national data protection agency (Commission Nationale de l’Informatique et des Libertés No 2014908). The Comite de Protection des Personnes Nord Ouest III waived the requirement for a written informed consent form. Patients were informed of the anonymous data collection in this study and were given the opportunity to express their opposition to participation.

### Statistical analyses

Categorical variables were described as frequencies and compared between the three groups using the χ^2^ test or Fisher’s exact test. Continuous variables were described by the median and compared using nonparametric tests (Kruskal–Wallis test). Comparison were repeated between JIA and the subgroup of *Kingella kingae* arthritis. Bonferroni correction for multiple comparisons was applied; this procedure set the threshold for significance at 0.025 (two-sided). Statistical analyses were performed using SAS statistical software (V.9.4; SAS institute).

### Ethics approval and consent to participate

The study was carried out in accordance with relevant national guidelines and regulations. The study was approved by the Institutional Review Board of Paris-North Hospitals, AP-HP (IRB no. 2013–84), the Comite de Protection des Personnes Nord Ouest III (2019-A02427-50) and the French national data protection agency (Commission Nationale de l’Informatique et des Libertés No 2014908). The Comite de Protection des Personnes Nord Ouest III waived the requirement for a written informed consent form. Patients were informed of the anonymous data collection in this study and were given the opportunity to express their opposition to participation.

## Results

**Figure 1:**
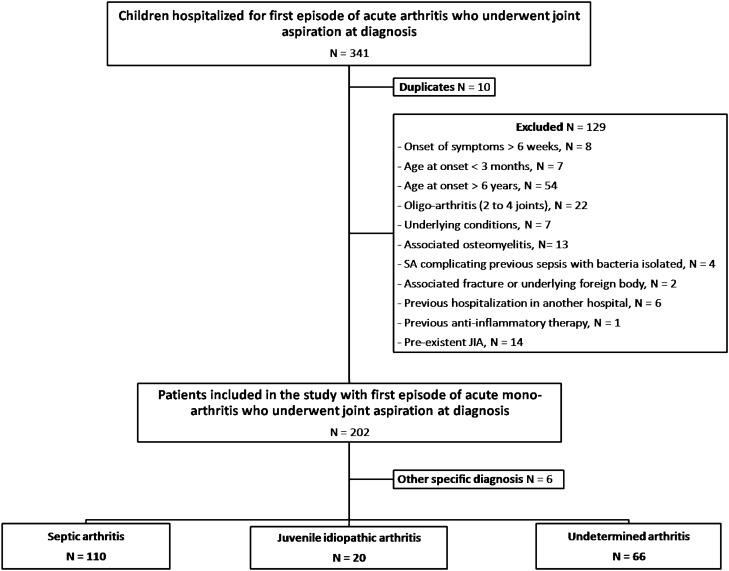
Flow chart.

Among the 341 eligible patients, 129 presented exclusion criteria and 6 patients had other specific diagnosis. We included and analyzed 196 patients: 110 patients (56.1%) had SA, 20 (10.2%) had JIA, and for 66 patients (33.7%) the arthritis remained of undetermined origin. The flow chart of patient’s selection is shown Fig. 1.

Demographic and clinical characteristics are detailed in Table 1. Patients with SA were significantly younger with a median age of 1.4 years-old [1.1–2.1], compared to patients with JIA (2.7 [1.8–3.6]) and UA (2.4 [1.5–4.0]), p < 0.001. 51.8% of patients with SA were boys, 40% of patients with JIA and 62.1% of UA, without significant difference. Concerning the topography of affected joints, arthritis of the knee was more frequent in JIA (70%) compared to SA (52.7%) and UA (42.4%), p = 0.01. Arthritis of the hip concerned 42.4% UA, 25% were related to JIA and 20% to SA. Fever preceding or present at admission concerned 58.3% of JIA patients, 79.4% septic patients and 75.6% UA, without statistic difference (p = 0.3).

**Table 1:** Clinical characteristics of children with septic arthritis, juvenile idiopathic arthritis and arthritis with no definitive diagnosis.

	According to group			
	JIA (N = 20)	SA (N = 110)	UA (N = 66)	p-value
**Age at diagnosis (years), median (IQR)**	2.7 [1.8–3.6]	1.4 [1.1–2.1]	2.4 [1.5–4.0]	< 0.001
**Male, % (n)**	40.0 (8)	51.8 (57)	62.1 (41)	0.17
**Onset in autumn or winter, %(n)**	45.0 (9)	50.9 (56)	37.9 (25)	0.24
**Affected joints,** **% (n)**				
Knee	70.0 (14)	52.7 (58)	42.4 (28)	0.01
Hip	25.0 (5)	20.0 (22)	42.4 (28)	
Ankle	5.0 (1)	10.0 (11)	4.6 (3)	
Other joints	0.0 (0)	17.3 (19)	10.6 (7)	
**Fever prior to admission or at admission, % (n/N)**	58.3 (7/12)	79.4 (50/63)	75.6 (31/41)	0.30

*SA* septic arthritis, *JIA* juvenile idiopathic arthritis, *UA* undetermined arthritis^a^, *IQR* interquartile range.

^a^Arthritis with no established association with infection that did not fulfil the classification criteria for JIA or other well-established diagnoses.

Results from blood and synovial analysis are presented in Table 2. JIA was associated with lower WBC counts (11.2 × 10^9^/L [10.0–13.6]) when compared to SA (13.2 × 10^9^/L [11.0–16.6]), p = 0.01. CRP level was significantly higher in SA with median CRP 37 mg/L [22–74] versus 27 [10–58] in JIA and 25 [12–40] in UA, p < 0.001. No significant difference was noted for fibrinogen level. JIA was associated with higher platelets level (444 × 10^9^ cells/L [322–554]) versus SA (366 × 10^9^ cells/L [295–490]) and UA (355 × 10^9^ cells/L [292–435]), p < 0.01. PCT did not significantly differ between groups, the high number of missing data has to be considered for this parameter. The leucocytes count in synovial fluid was significantly higher in SA (105,500 cells/mm^3^ [46,000–211,000]) when compared to UA (7295 cells/mm^3^ [210–72,000]) and JIA (42,000 cells/mm^3^ [6400–59,200]), p < 0.01. For all groups, leucocytes in synovial fluid were predominantly neutrophils.

**Table 2:** Biological characteristics of children with septic arthritis, juvenile idiopathic arthritis and arthritis with no definitive diagnosis.

Variable	JIA (n = 20)	SA (n = 110)	UA (n = 66)	p-value
**CRP (mg/L)**				
Median (IQR)	27 [10–58]	37 [22–74]	25 [12–40]	< 0.01
Min–max	5–134	5–246	5–205	
Missing value	N = 1	N = 15	N = 9	
**Fibrinogen (g/L)**				
Median (IQR)	4.2 [3.8–4.6]	5.3 [4.6–5.7]	4.6 [4.0–5.8]	0.03
Min–max	3.0–4.9	2.8–7.4	1.8–6.8	
Missing value	N = 12	N = 64	N = 36	
**PCT**				
Median (IQR)	0.05 [0–0.10]	0.05 [0–0.10]	0.2 [0–0.7]	0.28
Min–max	0–0.10	0–0.10	0–6.8	
Missing value	N = 18	N = 112	N = 56	
**WBC counts (× 10**^**9**^**/L)**				
Median (IQR)	11.2 [10.0–13.6]	13.2 [11.0–16.6]	12.0 [9.0–14.3]	0.01
Min–max	7.2–21.0	5.5–30.7	6.0–20.5	
Missing value	N = 5	N = 9	N = 10	
**Neutrophil counts (× 10**^**9**^**/L)**				
Median (IQR)	6.1 [4.3–9.2]	5.4 [4.3–8.6]	5.9 [4.1–7.6]	0.80
Min–max	2.2–4.3	0.9–23.1	1.9–14.2	
Missing value	N = 5	N = 43	N = 30	
**Haemoglobin (g/dL)**				
Median	11.4 [11.0–11.9]	10.9 [10.2–11.4]	11.8 [11.0–12.5]	< 0.01
Min–max	9.2–12.3	8.5–13.0	10.2–13.1	
Missing value	N = 11	N = 63	N = 39	
**Platelets (× 10**^**9**^**/L)**				
Median (IQR)	444 [322–554]	366 [295–490]	355 [292–435]	0.21
Min–max	277–591	172–1117	154–715	
Missing value	N = 2	N = 19	N = 13	
**Synovial WBC counts (cells/mm**^**3**^**)**				
Median (IQR)	42,000 [6400–59,200]	105,500 [46,000–211,000]	7295 [210–72,000]	< 0.001
Min–max	141–126,000	22–2,790,000	18–1,000,000	
Missing value	N = 11	N = 66	N = 40	
**Synovial WBC counts**				
≥ 50,000 cells/mm^3^, % (n/N)	44.4 (4/9)	72.7 (32/44)	34.6 (9/26)	< 0.01
**Synovial WBC counts**				
≥ 64,000 cells/mm^3^, % (n/N)	22.3 (2/9)	68.2 (30/44)	26.9 (7/26)	< 0.01
**Synovial neutrophiles** (%)				
Median (IQR)	76 [45–90]	92.5 [85.0–95.0]	81 [60–93]	0.01
Min–max	0–100	1.0–100.0	3–96	
Missing value	N = 11	N = 68	N = 45	
**Synovial lymphocytes** (%)				
Median (IQR)	10 [2–52]	2 [0–5]	8 [1–9]	0.09
Min–max	2–52	0–18	0–37	
Missing value	N = 17	N = 92	N = 56	

*SA* septic arthritis, *JIA* juvenile idiopathic arthritis, *UA* undetermined arthritis^a^, *IQR* interquartile range.

^a^Arthritis with no established association with infection, that did not fulfil the classification criteria for JIA or other well-established diagnoses.

Results concerning treatments and hospital stay are presented Table 3. Median length of hospital stay was 4 days in JIA [3–12] and UA [3–7], and 5 days [4–7] in SA, without significant difference. Antibiotics were administered to 95% of patients with JIA and 95.4% of UA, without significant difference compared to SA. In patients treated with antibiotics, the median duration of intravenous antibiotherapy was similar between groups with a median of 6 days in JIA [3–10] and SA [5–7], and 5 days [4–7] in UA, p = 0.66. Surgical arthrotomy and lavage was performed in 66.7% of patients with JIA, 79.6% of SA and 71.2% of UA, without significant difference.

**Table 3:** Follow-up children with septic arthritis, JIA and arthritis with no definitive diagnosis.

Variable	JIA (n = 20)	SA (n = 110)	UA (n = 66)	p-value
**Duration of hospital stay**				
Median (IQR)	4 [3–12]	5 [4–7]	4 [3–7]	0.07
Min–max	2–32	0–25	0–16	
Missing value	0	0	0	
**Antibiotic treatment, % (n)**	95.0 (19)	100.0 (110)	95.4 (62)	0.07
Missing value	0	0	1	
**Duration of IV antibiotic (if antibiotic)**				
Median (IQR)	6 [3–10]	6 [5–7]	5 [4–7]	0.66
Min–max	2–22	3–11	3–13	
Missing value	10	46	34	
**Type of surgery, % (n)**				
Arthroscopy or arthrotomy + irrigation	66.7 (12)	79.6 (82)	71.2 (42)	0.32
Joint puncture only	33.3 (6)	20.4 (21)	28.8 (17)	
Missing value	2	7	7	

*SA* septic arthritis, *JIA* juvenile idiopathic arthritis, *UA* undetermined arthritis^a^, *IQR* interquartile range.

^a^Arthritis with no established association with infection that did not fulfil the classification criteria for JIA or other well-established diagnoses.

Available data regarding microbiological explorations and identified microorganisms in SA are shown in Table 4. The first causative agent of SA in our cohort was KK, representing 82.7% (91/110) of SA, the other minority of patients had *Streptococcus* (7.4%; 8/110) or *Staphylococcus aureus* (2.7%; 3/110) or other germs. Among the 110 cases of SA explored by standard culture, only 26 (23.6%) were positive. Specific PCR for KK was performed on 100 patients and was positive for 89% of them.

**Table 4:** Septic arthritis: description of positive microbiological examinations and identified microorganisms.

	N = 110	
	n	%
**Positive microbiological examination**		
Synovial fluid—direct microscopic examination	4/77	5.2
Synovial fluid—culture	26/110	23.6
Synovial fluid—PCR-KK	89/100	89.0
Synovial fluid—PCR-16S	3/12	25.0
Blood—culture	8/70	11.4
**Identified microorganism**		
*Kingella kingae*	91	82.7
Group A *Streptococcus*	4	3.7
*Streptococcus pneumoniae*	4	3.7
*Staphylococcus aureus*	3	2.7
Salmonella	2	1.8
Coagulase negative *Staphylococcus*	2	1.8
Group B meningococcus,	2	1.8
*Haemophilus *spp.	1	0.9
*Candida tropicalis*	1	0.9

*PCR-KK:* PCR for *Kingella kingae* detection.

Finally, we compared clinical and biological features focusing on patients with SA due to KK and their comparison to JIA, the data are shown Table 5. Patients with KK-SA were significantly younger with median age of 1.3 years-old [1.1–1.6], compared to JIA 2.7 [1.8–3.6], p < 0.001. There was no difference in localization of arthritis; 55% of KK-SA and 70% of JIA had monoarthritis of the knee, 17.5% of KK-SA and 25% of JIA had hip monoarthritis. Concerning biological data in blood, only fibrinogen level was significantly lower in JIA 4.2 [3.8–4.6] compared to SA 5.1 [4.5–5.6], p < 0.01. No difference was observed with respect to CRP and WBC count. Synovial WBC count was significantly higher in KK-SA 108,000/mm^3^ [64,000–210,000] compared to JIA with 42,000/mm^3^ [6400–59,200], p < 0.01.

**Table 5:** Characteristics and care of children with juvenile idiopathic arthritis and septic arthritis due to *Kingella kingae*.

	JIA (n = 20)	SA – KK (n = 91)	p-value
**Age at diagnosis (years)**			
Median (IQR)	2.7 [1.8–3.6]	1.3 [1.1–1.6]	< 0.001
**Male, % (n)**	40.0 (8)	52.8 (48)	0.30
**Onset in autumn or winter, % (n)**	45.0 (9)	52.8 (48)	0.53
**Affected joints, % (n)**			
Knee	70.0 (14)	55.0 (50)	0.12
Hip	25.0 (5)	17.5 (16)	
Ankle	5.0 (1)	8.8 (8)	
Other joints	0.0 (0)	19.7 (17)	
**Fever prior to admission or at admission, % (n/N)**	58.3 (7/12)	75.5 (37/49)	0.29
**CRP (mg/L)**			
Median (IQR)	27 [10–58]	34 [21–63]	0.27
Min–max	5–134	5–133	
Missing value	N = 1	N = 12	
**Fibrinogen (g/L)**			
Median (IQR)	4.2 [3.8–4.6]	5.1 [4.5–5.6]	< 0.01
Min–max	3.0–4.9	2.8–6.5	
Missing value	N = 12	N = 55	
**PCT**			
Median (IQR)	0.05 [0–0.10]	0.05 [0–0.10]	0.99
Min–max	0–0.10	0–0.10	
Missing value	N = 18	N = 83	
**WBC counts (× 10**^**9**^**/L)**			
Median (IQR)	11.2 [10.0–13.6]	13.2 [11.1–16.2]	0.05
Min–max	7.2–21.0	7.8–27.9	
Missing value	N = 5	N = 6	
**Neutrophil counts (× 10**^**9**^**/L)**			
Median (IQR)	6.1 [4.3–9.2]	5.3 [4.3–8.0]	0.37
Min–max	2.2–4.3	1.8–1.5	
Missing value	N = 5	N = 37	
**Haemoglobin (g/dL)**			
Median	11.4 [11.0–11.9]	11.1 [10.4–11.4]	0.27
Min–max	9.2–12.3	9.0–12.8	
Missing value	N = 11	N = 55	
**Platelets (× 10**^**9**^**/L)**			
Median (IQR)	444 [322–554]	386 [322–498]	0.48
Min–max	277–591	221–1117	
Missing value	N = 2	N = 15	
**Synovial WBC counts (cells/mm**^**3**^**)**			
Median (IQR)	42,000 [6400–59,200]	108,000 [64,000–210,000]	< 0.01
Min–max	141–126,000	940–2,790,000	
Missing value	N = 11	N = 54	
**Synovial WBC counts**			
≥ 50,000 cells/mm^3^, % (n)	44.4 (4/9)	78.4 (29/37)	0.09
**Synovial WBC counts**			
≥ 64,000 cells/mm^3^, % (n)	22.3 (2/9)	75.7 (28/37)	< 0.01
**Synovial neutrophil counts (%)**			
Median (IQR)	76 [45–90]	93 [84–95]	0.04
Min–max	0–100	1–100	
Missing value	N = 11	N = 55	
**Synovial lymphocytes counts (%)**			
Median (IQR)	10 [2–52]	3 [2–6]	0.20
Min–max	2–52	0–18	
Missing value	N = 17	N = 77	

## Discussion

In our study, patients’ baseline clinical and biological data did not allow to reliably distinguish JIA from SA in young children less than 6 years of age with acute monoarthritis. This observation is in line with a previous study on a smaller cohort^20^. Similarly, in an adult population, a retrospective study did not identify any distinction between septic and aseptic arthritis concerning characteristics of patients at baseline, fever and serum laboratory parameters^21^.

As for the composition of our cohort, it was predominantly AS (56.1%), with JIAs representing only 10.2% of our patients. Among SA, 82% was KK-SA. The estimated incidence of bone and joint infections in children in France is 10 per 100,000 children, with KK accounting for 30% of bone and joint infections in children and up to 80% in children younger than 4 years of age^5,7,9,10,28^. Compared to the literature, our cohort had a higher rate of SA, with KK being implicated in the vast majority of cases. This could be explained by a selection bias, since we focused on children below the age of 6 years. In addition, we only included patients with mono-arthritis in need of hospitalization and having undergone joint puncture. These criteria may have led to an enhanced selection of patients with a high probability of SA and an exclusion of patients with non-septic arthritis, such as JIA with an oligo/polyarticular presentation. As involvement of multiple joints is exceptional in SA, patient with oligo or polyarthritis are generally referred primarily to outpatient pediatric rheumatology services of our accredited reference center for rare inflammatory and systemic rheumatological diseases. The JIA patients included in this study are thus not representative of a general JIA population. Our results must be interpreted in light of our inclusion criteria and the objective of the study, which is to focus on acute monoarthritis in young children.

Concerning the topography of arthritis in our study, knee was the most frequent localization in both SA and JIA. This is in accordance with observations from the literature showing that early-onset JIA manifests as initial monoarthritis in 70% of cases, mostly in the knee^29,30^. In contrast, mono-arthritis of the hip accounted for 25% of JIA patients, which is surprisingly high given that hip arthritis is not a common localization of oligoarticular JIA, especially in the early stages of the disease^30,31^. Arthritis of hip at onset has been described in psoriatic arthritis but remains rare, concerning only 5–10% of patients^32,33^. It is important to note that among the 6 patients with monoarthritis of the hip, the final diagnosis during the follow-up was of systemic JIA in 4 patients, polyarticular RF negative-JIA (n = 2). Though hip arthritis has been described in 20–40% of systemic JIA, it is generally observed after 1–6 years of evolution and occurs associated to polyarticular manifestations^34^. The difference between the observations from general JIA and results from our study cohort may be explained by our inclusion criteria selecting a specific population of patients in whom the diagnosis of JIA was not obvious at the earliest stage of arthritis given the clinical presentation of monoarthritis. Our data indicate that in current clinical practice, the JIA subgroups that present a diagnostic problem at the onset of monoarthritis before the age of 6 years, and for which a joint puncture is performed to exclude septic arthritis, are children with oligoarticular JIA and children with systemic JIA with hip arthritis.

Our results showed a significant difference in CRP levels, hemoglobin and WBC counts between groups, but with a large area of overlap. Similarly, synovial WBC counts were significantly higher in SA. A study in adult patients reported a difference on synovial WBC count higher in the septic group, with the threshold of 64 × 10^3^ cells/mm^3^ having the highest combined sensitivity (40%) and specificity (90%)^21^. In children, a previous study suggested that a WBC count higher than > 50 × 10^3^ cells/mm^3^ could allow distinction of 85% of the septic arthritis patients^20^. Using a larger cohort, we found WBC count > 50 × 10^3^ cells/mm^3^ in 72.7% of patients with SA, more frequent compared to JIA (44.4%) and UA (34.6%). This parameter with threshold of WBC count > 64 × 10^3^ cells/mm^3^ was statistically different between KK-SA (75.7%) and JIA (22.3%). Yet, it is to note that synovial WBC count may not be robust enough to implement it into the pediatric clinical practice. Indeed, an appropriate estimation of WBC count in synovial fluids was available for only 40.3% (79/196) of our samples. The detailed macroscopic description of synovial fluids was available for 90/196 (45.9%) of samples, 20 (22.2%) of which were described as clotted and 34 (37.8%) as hemorrhagic. Clotting and coagulation perturb the cell counts or may even render counting impossible. The variety of macroscopic aspects of synovial fluids may explain why technicians encounter problems when performing and/or documenting synovial cell count. Thus, high WBC counts in joint aspiration may incite the clinician to orientate the diagnosis towards SA but is not reliable and robust enough to differentiate between SA, JIA and other forms of non-septic arthritis.

Positivity of standard bacterial cultures was quite low in our population with positive results of synovial culture only for 26/110 (23.6%) patients with SA, underlying previously reported poor sensitivity of culture to detect SA. This result might be, at least partially, explained by KK being the prominent causative agent of SA in young children, leading to foiled standard cultures because of hard-to-grow bacteria^9,12^. In recent years the use of KK specific real-time PCR has markedly improved the etiological diagnosis of septic arthritis, with positivity up to 6 days after initiation of antibiotics^9^. In our study cohort 89/91 (97.8%) of KK infections had a positive PCR. This observation emphasizes that, at least in pediatric populations, genetic techniques may increase the sensitivity to detect an infectious agent.

The absence of a reliable diagnostic test for the differentiation of JIA and other arthritis impacts on treatments, especially in pediatric populations. The actual European recommendations in case of suspicion of SA, is to introduce probabilistic antibiotherapy and to perform joint lavage^26,35,36^. Indeed, among patients with JIA and UA we found a high percentage of retrospectively unnecessary therapy considering the final diagnosis. In our study population, 95% of patients with JIA and 95.4% of patients with UA received probabilistic intravenous antibiotics. When SA was not confirmed yet, but no argument for another diagnosis was available, probabilistic antibiotherapy was often pursued orally until a follow-up appointment with an orthopedist. Unfortunately, the available documentation did not allow us to evaluate the total duration of exposure to oral antibiotics in these patients. In the actualized practice of our center, it is now common to stop the antibiotherapy if culture and KK-PCR are negative in the absence of antibiotics’ exposure preceding the joint’s aspiration. In order to decrease unnecessary antibiotic exposure, there is a need for new diagnosis methods that allow early differentiation between septic and non-septic acute arthritis. Analyses of synovial fluids using proteomics or other OMIC techniques may in the near future allow to discover and develop clinically relevant biomarkers^37–39^.

Surgical treatment, such as open or needle arthrotomy and irrigation (lavage), can be necessary for treatment of SA^12,19,23–25,35,36,40,41^. In the SA group 79.6% of patients underwent arthrotomy. A review on bone and joint infections due to KK reports an arthrotomy only in 131 over 566 cases reviewed (23.1%)^6^. A bigger French national cohort based on 2911 children with bone and joint infections reported an arthrotomy rate of 59% similar to our data^7^. In our study cohort, 66.7% of patients with JIA and 71.2% with UA also underwent arthrotomy-lavage. These surgical procedures require general anesthesia and may induce prolonged duration of hospitalization, bed rest and complications. Therefore, it should ideally be reserved exclusively for SA patients. Thus, in absence of reliable markers to differentiate SA from acute non-septic arthritis, young patients with monoarticular JIA are exposed to invasive surgical treatments early on the course of the disease.

In accordance with previous studies, we found that acute arthritis remained undifferentiated for 1/3 of children presenting at the emergency ward for acute arthritis^27^. The clinical and biological profile of those patients with UA seems to be closer to the profile of patients with JIA than with SA, unless they have lower platelets and synovial WBC counts. Thus, the biological markers currently in use do not differentiate between these two groups and only evolution can differentiate them. The availability of reliable diagnostic biomarkers, using blood or joint puncture fluids, would significantly reduce the time to diagnosis and allow referral of JIA patients to a specialist for early initiation of specific treatment. Thus, ideal biomarkers should not only be able to differentiate between acute SA and acute non-septic arthritis but should also be able to identify JIA in relation to other forms of non-septic arthritis.

## Conclusions

In children less than 6 years of age with acute mono-arthritis, the clinical and biological parameters currently used do not reliably differentiate between JIA, AS and UA. JIA subgroups that present a diagnostic problem at the onset of monoarthritis before the age of 6 years, are oligoarticular JIA and systemic JIA with hip arthritis. The development of new biomarkers will be required to distinguish JIA and AS caused by *Kingella kingae* in these patients.

## Supplementary Information

Supplementary Table 1.

## Data Availability

All data generated or analyzed during this study are included in this published article.

## Abbreviations

JIA: Juvenile idiopathic arthritis
SA: Septic arthritis
UA: Undetermined arthritis
CRP: C-reactive protein
PCT: Pro-calcitonin
IV: Intra-venous
IQR: Interquartile range

## References

[1] Crayne CB, Beukelman T (2018). Juvenile idiopathic arthritis. Pediatr. Clin. North Am..

[2] Prakken B, Albani S, Martini A (2011). Juvenile idiopathic arthritis. Lancet Lond. Engl..

[3] Petty RE (2004). International League of Associations for Rheumatology classification of juvenile idiopathic arthritis: second revision, Edmonton, 2001. J. Rheumatol..

[4] Thierry S, Fautrel B, Lemelle I, Guillemin F (2014). Prevalence and incidence of juvenile idiopathic arthritis: A systematic review. Joint Bone Spine.

[5] Yagupsky P (2015). Kingella kingae: Carriage, transmission, and disease. Clin. Microbiol. Rev..

[6] Al-Qwbani M, Jiang N, Yu B (2016). *Kingella**kingae*-associated pediatric osteoarticular infections: An overview of 566 reported cases. Clin. Pediatr. (Phila.).

[7] Laurent E (2016). Évolution des infections ostéo-articulaires (IOA) en France après mise en place des centres de références des IOA complexes (CRIOAC): PMSI 2008 versus 2013. Rev. d’Épidémiologie Santé Publique.

[8] Okubo Y, Nochioka K, Marcia T (2017). Nationwide survey of pediatric septic arthritis in the United States. J. Orthop..

[9] Ilharreborde B (2009). New real-time PCR-based method for *Kingella**kingae* DNA detection: Application to samples collected from 89 children with acute arthritis. J. Clin. Microbiol..

[10] Gravel J (2017). Association between oropharyngeal carriage of *Kingella**kingae* and osteoarticular infection in young children: A case–control study. CMAJ Can. Med. Assoc. J..

[11] Lorrot M (2011). Antibiothérapie des infections ostéo-articulaires de l’enfant: ce qui a changé. Arch. Pédiatr..

[12] Ferroni A (2012). Prospective survey of acute osteoarticular infections in a French paediatric orthopedic surgery unit: Acute osteoarticular infections in children. Clin. Microbiol. Infect..

[13] Petit L (2016). Facteurs de risque d’hospitalisation prolongée pour infection ostéo-articulaire pédiatrique en France à partir du PMSI 2013. Rev. d’Épidémiologie Santé Publique.

[14] Janeway CA (1989). Approaching the asymptote? Evolution and revolution in immunology. Cold Spring Harb. Symp. Quant. Biol..

[15] Silva-Gomes S, Decout A, Nigou J, Parnham M (2014). Pathogen-associated molecular patterns (PAMPs). Encyclopedia of Inflammatory Diseases.

[16] Fox A, Fox K, Christensson B, Harrelson D, Krahmer M (1996). Absolute identification of muramic acid, at trace levels, in human septic synovial fluids in vivo and absence in aseptic fluids. Infect. Immun..

[17] Chen T (2003). Bacterial components in the synovial tissue of patients with advanced rheumatoid arthritis or osteoarthritis: Analysis with gas chromatography-mass spectrometry and pan-bacterial polymerase chain reaction: Bacterial components in rheumatoid arthritis. Arthritis Care Res..

[18] Lyon RM, Evanich JD (1999). Culture-negative septic arthritis in children. J. Pediatr. Orthop..

[19] Chaput C, Boneca IG (2007). Peptidoglycan detection by mammals and flies. Microbes Infect..

[20] Aupiais C (2017). Arthritis in children: Comparison of clinical and biological characteristics of septic arthritis and juvenile idiopathic arthritis. Arch. Dis. Child..

[21] Borzio R (2016). Predictors of septic arthritis in the adult population. Orthopedics.

[22] Kocher MS, Mandiga R, Zurakowski D, Barnewolt C, Kasser JR (2004). Validation of a clinical prediction rule for the differentiation between septic arthritis and transient synovitis of the hip in children. J. Bone Joint Surg. Am..

[23] Agout C, Lakhal W, Fournier J, de Bodman C, Bonnard C (2015). Traitement arthroscopique des arthrites septiques du genou de l’enfant. Rev. Chir. Orthop. Traumatol..

[24] Johns B, Loewenthal M, Ho E, Dewar D (2017). Arthroscopic versus open treatment for acute septic arthritis of the knee in children. Pediatr. Infect. Dis. J..

[25] Wirtz D, Marth M, Miltner O, Schneider U, Zilkens K (2001). Septic arthritis of the knee in adults: Treatment by arthroscopy or arthrotomy. Int. Orthop..

[26] Grimprel E (2008). Infections ostéoarticulaires: Propositions thérapeutiques du Groupe de Pathologie Infectieuse Pédiatrique (GPIP) de la Société Française de Pédiatrie. Arch. Pédiatr..

[27] Aupiais C (2015). Aetiology of arthritis in hospitalised children: An observational study. Arch. Dis. Child..

[28] Wong M, Williams N, Cooper C (2020). Systematic review of *Kingella**kingae* musculoskeletal infection in children: Epidemiology, impact and management strategies. Pediatr. Health Med. Ther..

[29] Stoll ML, Nigrovic PA, Gotte AC, Punaro M (2011). Clinical comparison of early-onset psoriatic and non-psoriatic oligoarticular juvenile idiopathic arthritis. Clin. Exp. Rheumatol..

[30] Job-Deslandre C (2007). Arthrites juvéniles idiopathiques. EMC Appar. Locomot..

[31] Rostom S, Amine B, Bensabbah R, Abouqal R, Hajjaj-Hassouni N (2008). Hip involvement in juvenile idiopathic arthritis. Clin. Rheumatol..

[32] Huemer C (2002). Patterns of joint involvement at onset differentiate oligoarticular juvenile psoriatic arthritis from pauciarticular juvenile rheumatoid arthritis. J. Rheumatol..

[33] Stoll ML (2006). Patients with juvenile psoriatic arthritis comprise two distinct populations. Arthritis Rheumatol..

[34] Batthish M, Feldman BM, Babyn PS, Tyrrell PN, Schneider R (2011). Predictors of hip disease in the systemic arthritis subtype of juvenile idiopathic arthritis. J. Rheumatol..

[35] E. Grimpel, M. L. Antibiotic therapy of bone and joint infections (BJI) in children: Propositions of the Groupe de Pathologie Infectieuse Pédiatrique (GPIP). *Arch. Pédiatr.* (2016).

[36] Lorrot M (2017). Antibiotic therapy of bone and joint infections in children: Proposals of the French Pediatric Infectious Disease Group. Arch. Pédiatr..

[37] Nziza N (2019). Synovial-fluid miRNA signature for diagnosis of juvenile idiopathic arthritis. Cells.

[38] Kessel C (2018). Proteomics in chronic arthritis—will we finally have useful biomarkers?. Curr. Rheumatol. Rep..

[39] Zhang F (2019). Defining inflammatory cell states in rheumatoid arthritis joint synovial tissues by integrating single-cell transcriptomics and mass cytometry. Nat. Immunol..

[40] Fabry G, Meire E (1983). Septic arthritis of the hip in children: Poor results after late and inadequate treatment. J. Pediatr. Orthop..

[41] Christiansen P, Frederiksen B, Glazowski J, Scavenius M, Knudsen FU (1999). Epidemiologic, bacteriologic, and long-term follow-up data of children with acute hematogenous osteomyelitis and septic arthritis: A ten-year review. J. Pediatr. Orthop. Part B.

