# Supplementary material for: Acute monoarthritis in young children: comparing the characteristics of patients with juvenile idiopathic arthritis versus septic and undifferentiated arthritis
Source: Sci Rep. 2021 Feb 9;11:3422. doi: 10.1038/s41598-021-82553-1 (PMC7873238; doi:10.1038/s41598-021-82553-1)
Supplement: Supplementary file 1 — Supplementary Table 1. [file 41598_2021_82553_MOESM1_ESM.docx]

**Acute monoarthritis in young children - comparing the characteristics of patients with juvenile idiopathic arthritis versus septic and undifferentiated arthritis**

# Authors: Marion Thomas^1,2^, Stephane Bonacorsi^3,4^, Anne-Laure Simon^3,5^, Cindy Mallet^3,5^, Mathie Lorrot^6^, Albert Faye^1,3^, Glory Dingulu^1^, Marion Caseris^1^, Ivo Gomperts Boneca^2^, Camille Aupiais*^7,8^, Ulrich Meinzer*^1,2,3,9^

**Affiliations:**

^1^Department of General Pediatrics, Pediatric Internal Medicine, Rheumatology and Infectious Diseases, National Reference Centre for Rare Pediatric Inflammatory Rheumatisms and Systemic Autoimmune diseases RAISE, Robert Debré University Hospital, Assistance Publique - Hôpitaux de Paris, Paris, France.

^2^Institut Pasteur, Biology and Genetics of Bacterial Cell Wall Unit, Paris; CNRS UMR2001, Paris, France; INSERM, Equipe Avenir, Paris, France.

^3^Université de Paris, Paris, France.

^4^Department of Microbiology, Robert Debré University Hospital, Assistance Publique - Hôpitaux de Paris, Paris, France.

^5^PediatricOrthopedicDepartment, Robert Debré University Hospital, Assistance Publique - Hôpitaux de Paris, Paris, France.

^6^Pediatric Department - Division of infectious diseases, Armand Trousseau Hospital, Assistance Publique - Hôpitaux de Paris, Paris, France.

^7^ Pediatric Emergency Department, Jean Verdier Hospital, Assistance Publique - Hôpitaux de Paris, Paris 13 University, Bondy, France.

^8^ INSERM, U1138, Equipe 22, Centre de Recherche des Cordeliers, Paris, France.

^9^ Centre de recherche sur l'inflammation, UMR1149 INSERM et Université de Paris, France.

**Supplementary data – table 1:** clinical and biological characteristics of patients, description and comparison of inclusion cohorts.

|  | **Cohort 2015-2018**  **(n=96)** |  | **Cohort 2008-2009**  **(n=100)** |  | **p-value** |
| --- | --- | --- | --- | --- | --- |
| **Septic arthritis,** %(n) | 54.2 (52) |  | 58.0 (58) |  | 0.71 |
| **Juvenile idiopathic arthritis,** %(n) | 9.4 (9) |  | 11.0 (11) |  |  |
| **Undetermined arthritis,** %(n) | 36.4 (35) |  | 31.0 (31) |  |  |
| **Microbiology of septic arthritis,** % (n) |  |  |  |  |  |
| *Kingella kingae* | 78.8 (41) |  | 86.2 (50) |  |  |
| Other germs | 81.2 (11) |  | 13.8 (8) |  |  |
| **Age at diagnosis (years)**  Median (IQR) | 1.6 [1.1-2.9] |  | 1.7 [1.2-3.1] |  | 0.53 |
| **Male,** % (n) | 54.2 (52) |  | 54.0 (54) |  | 0.98 |
| **Onset in autumn or winter,** %(n) | 49.0 (47) |  | 43.0 (43) |  | 0.40 |
| **Affected joints,** % (n) |  |  |  |  | 0.31 |
| Knee | 48.0 (46) |  | 54.0 (54) |  |  |
| Hip | 26.0 (25) |  | 30.0 (30) |  |  |
| Ankle | 8.3 (8) |  | 7.0 (7) |  |  |
| Other joints | 17.7 (17) |  | 9.0 (9) |  |  |
| **CRP (mg/L)**  Median (IQR)  Min-max | 32 [18-52]  5-162 |  | 33 [19-64]  5-246 |  | 0.56 |
| Missing value | N = 19 |  | N = 6 |  |  |
| **WBC counts(x10^9^/L)**  Median (IQR)  Min-max | 12.8 [10.8-16.1]  5.5-29.8 |  | 12.6 [9.9-15.0]  6.1-30.7 |  | 0.56 |
| Missing value | N = 16 |  | N = 4 |  |  |
| **Neutrophil counts (x10^9^/L)**  Median (IQR)  Min-max | 5.7 [4.3-7.7]  0.9-23.1 |  | 6.5 [4.1-9.2]  2.0-22.7 |  | 0.40 |
| Missing value | N = 33 |  | N = 45 |  |  |
| **Haemoglobin (g/dL)**  Median  Min-max | 11.2 [10,7-11.9]  8.5-13.1 |  | 11.0 [10.0 -12.0]  10.0 -13.0 |  | 0.49 |
| Missing value | N = 23 |  | N = 90 |  |  |
| **Platelets (x10^9^/L)**  Median (IQR)  Min-max | 387 [298-505]  172-1 117 |  | 362 [295-469]  154-702 |  | 0.50 |
| Missing value | N = 23 |  | N = 11 |  |  |
